# Insect and fish cell lines support replication and serial passage of covert mortality nodavirus

**DOI:** 10.3389/fmicb.2026.1858574

**Published:** 2026-08-05

**Authors:** Zhaoxi Wang, Zhanqing Sun, Tingting Xu, Na Wang, Qingli Zhang

**Affiliations:** 1 State Key Laboratory of Mariculture Biobreeding and Sustainable Goods, Key Laboratory of Maricultural Organism Disease Control, Ministry of Agriculture, Qingdao Key Laboratory of Mariculture Epidemiology and Biosecurity, Yellow Sea Fisheries Research Institute, Chinese Academy of Fishery Sciences, Qingdao, Shandong, China; 2 Laboratory for Marine Fisheries Science and Food Production Processes, Laoshan Laboratory, Qingdao, Shandong, China

**Keywords:** covert mortality nodavirus (CMNV), viral covert mortality disease (VCMD), cross-species transmission, serial passage, viral replication, virus-host interactions

## Abstract

Covert mortality nodavirus (CMNV) is the pathogen causing viral covert mortality disease (VCMD) in cultured shrimp, and cross-species transmission is an important characteristic of this virus. The lack of stable, continuously passable shrimp cell lines has long been a bottleneck for in-depth studies on CMNV pathogenesis, virus-host interactions, and antiviral strategies. In this study, we systematically evaluated the infectivity, replication kinetics, and serial passage stability of CMNV in eight cell lines of different origins, including three insect cell lines (Sf21, C6/36, and S2) and five fish cell lines (EMRB, EPC, ZF4, TiB, and GiCB). Based on CPE observations and viral RNA replication profiles across three blind passages, cell lines exhibiting both obvious CPE and viral RNA replication in at least two passages were classified as susceptible, including EPC (P1 and P3), TiB (P1 and P3), and S2 (P1-P3). ZF4 (P1-P3) and C6/36 (P1 and P3), despite lacking obvious CPE, supported viral RNA replication across multiple passages and were therefore tentatively designated as atypically susceptible. In contrast, Sf21 and EMRB showed no detectable viral RNA replication in any of the three passages, while GiCB supported replication only at P1; these three cell lines were accordingly classified as non-susceptible. These findings identify multiple non-shrimp cell lines that support stable CMNV replication at the RNA level and serial passage, providing useful experimental systems for studies of viral pathogenesis, host interactions, diagnostic development, and antiviral product screening.

## 1 Introduction

Covert mortality nodavirus (CMNV), a member of the family Nodaviridae, causes viral covert mortality disease (VCMD) in cultured shrimp (Zhang et al., 2014; Liu et al., 2022; Yao et al., 2022). CMNV was first reported in Guangxi and Hainan provinces of China and subsequently spread to more than ten coastal provinces in 2012. Annual positive detection rates ranged from 10.04 to 45.93% during 2013–2022 (Li et al., 2019; Zhao et al., 2024). CMNV has also been detected in Thailand, Vietnam, Mexico, Ecuador, and several other countries (Flegel, 2014; Pooljun et al., 2016; Varela-Mejías, 2018). In 2023, the World Organisation for Animal Health (WOAH) classified CMNV infection as an emerging aquatic disease (WOAH, 2023). Remarkably, CMNV exhibits an extremely broad host range. The virus infects major cultured shrimp species and crosses species barriers to infect jellyfish (Hao, 2020), sea cucumbers (Wang et al., 2021a; Wang et al., 2021b), and several teleost fish species, including *Paralichthys olivaceus*, *Acanthopagrus schlegelii*, *Carassius auratus* (Wang, 2020), *Larimichthys polyactis*, and *L. crocea* (Xu et al., 2021; Xu et al., 2022). CMNV also undergoes both vertical (via sperm and eggs in *Exopalaemon carinicauda*) and horizontal transmission (Liu et al., 2017). Potential reservoir hosts include copepods, *Corophium acherusicum*, hermit crabs, and *Themisto gracilipes* (Zhang et al., 2017; Liu et al., 2018).

Recently, a study published in *Nature Microbiology* reported for the first time that CMNV can infect humans and is associated with persistent ocular hypertension-viral anterior uveitis (POH-VAU) in humans (Liu et al., 2026). The same study also demonstrated CMNV infection in human corneal endothelial cells (HCEC) and African green monkey kidney (Vero) cells. These findings highlight the zoonotic potential of CMNV and expand its known host range. However, the molecular mechanisms that govern CMNV cross-species infections remain largely unknown. Several studies have investigated CMNV infections in cultured cell lines. Our previous work shows that CMNV infection induces cytopathic effects in EPC cells, which indicates that these cells are susceptible to infection (Xu et al., 2022). Zhao et al. used reverse genetics to construct a recombinant CMNV that expresses enhanced green fluorescent protein (EGFP). The recombinant virus infected both Sf9 cells and brown-marbled grouper fin cells and provided a visual tool for studies of CMNV gene function (Zhao et al., 2026). However, these studies examined only a limited number of cell lines. A systematic evaluation of CMNV infectivity, replication kinetics, and passage stability in different animal cell lines remains unavailable. This gap has limited the development of robust *in vitro* models for CMNV research.

In this study, we systematically evaluated CMNV infectivity and replication in eight cell lines of different origins, including three insect cell lines (*Spodoptera frugiperda* 21, Sf21; *Aedes albopictus* clone C6/36, C6/36; and Schneider 2, S2) and five fish cell lines (*Epinephelus moara* brain cell, EMRB; *Epithelioma papulosum cyprini*, EPC; zebrafish embryo fibroblast cell, ZF4; tilapia brain cell, TiB; and Gibel carp brain cell, GiCB). We also assessed cytopathic effects and viral stability during serial passage. Our aim was to identify CMNV-susceptible cell lines and establish experimental systems that support studies of CMNV gene function, diagnostic development, and antiviral intervention.

## 2 Materials and methods

### 2.1 Virus preparation

The crude CMNV extract was obtained from CMNV-positive *Artemia*. These extracts were used to infect *Litopenaeus vannamei* for viral amplification. After the infected shrimps developed typical clinical symptoms, their tissues were collected and homogenized. The homogenate was filtered through a nylon sieve and subjected to ultracentrifugation (Ultracentrifuge CP100WX, Hitachi, Japan) to obtain a crude CMNV preparation. The virus solution was divided into aliquots and stored at −80 °C for subsequent cell infection experiments.

### 2.2 Source and culture conditions of cell lines

Eight cell lines were used in this study, including three insect cell lines and five fish cell lines (Table 1). All cells were cultured in specific media under optimized conditions. Cells were routinely subcultured in 75 cm^2^ cell culture flasks (Corning Inc., Corning, NY, USA) at a passage ratio of 1:2 to 1:4, with two to three passages per week. All cells used in the experiments were in the logarithmic growth phase, and cell viability exceeded 95%, as determined by trypan blue staining.

**Table 1:** Origin and culture conditions of the cell lines used in this study.

Cell line	Origin	Source	Culture medium	Culture conditions
Sf21	*Spodoptera frugiperda* ovary	Professor Ouyang Songying, Fujian Normal University	SIM-SF (serum-free)	28 °C
C6/36	*Aedes albopictus*	Wuhan Procell Life Science & Technology Co., Ltd.	Eagle’s MEM + 10% FBS + 2% penicillin–streptomycin	28 °C 5% CO₂
S2	*Drosophila melanogaster*	Wuhan Procell Life Science & Technology Co., Ltd.	Schneider’s Drosophila Medium + 10% FBS + 2% penicillin–streptomycin	28 °C
EMRB	*Epinephelus moara* brain	Professor Wang Na, Yellow Sea Fisheries Research Institute	Leibovitz’s L-15 + 10% FBS + 2% penicillin–streptomycin	28 °C
EPC	*Epithelioma papulosum cyprini*	Professor Zhang Yong’an, Huazhong Agricultural University	M199 + 10% FBS + 2% penicillin–streptomycin	28 °C 5% CO₂
ZF4	Zebrafish embryo fibroblast	Professor Zhang Yong’an, Huazhong Agricultural University	DMEM/F12 (1:1) + 10% FBS + 2% penicillin–streptomycin	28 °C 5% CO₂
TiB	Tilapia brain	Professor Zeng Weiwei, Foshan University of Science and Technology	M199 + 10% FBS + 2% penicillin–streptomycin	28 °C 5% CO₂
GiCB	Gibel carp brain	Professor Fan Yuding, Yangtze River Fisheries Research Institute	M199 + 10% FBS + 2% penicillin–streptomycin	28 °C 5% CO₂

### 2.3 Virus infection and sample collection

Each cell line was seeded into 35 mm cell culture dishes (Corning, USA) at an appropriate density (approximately 5 × 10^5^ cells/dish) and incubated overnight under the appropriate culture conditions to allow cell attachment. Before infection, the old medium was aspirated and replaced with maintenance medium (infection medium) containing 2% FBS. For initial susceptibility screening across eight cell lines, 50 μL of virus extract was added to each dish, whereas an equal volume of phosphate-buffered saline (PBS, pH 7.4) was added to the control group. Following the identification of sensitive cell lines, viral titers were determined by TCID50 assays, and subsequent replication kinetics experiments were performed at a standardized MOI to quantitatively compare replication efficiency. After infection, cells were incubated under the corresponding culture conditions (28 °C, with or without CO₂ depending on cell type). Samples were collected at 6 h post-infection (hpi) and at 12, 24, 48, 72, and 96 hpi. Three replicate dishes were included for each time point. At each sampling time point, the supernatant was carefully aspirated, and the cells were washed three times with pre-cooled PBS. Subsequently, 1 mL of TRIzol reagent (Invitrogen, Carlsbad, CA, USA) was added to each dish. The cells were lysed, collected by repeated pipetting, transferred to RNase-free 1.5 mL centrifuge tubes, and stored at −80 °C until RNA extraction.

### 2.4 Serial viral passage

To evaluate the adaptive capacity of CMNV for continuous passage in different cell lines, a three-generation blind-passage experiment was performed. The specific steps are as follows: First passage (P1): Cells were seeded into 35 mm culture dishes and allowed to adhere overnight. Viral infection was then performed as described in Section 2.3. Briefly, 50 μL of virus extract was added to each dish. At 72 hpi, cell cultures were collected. Cells and supernatant were thoroughly mixed and transferred to 15 mL centrifuge tubes. The samples were then subjected to three freeze–thaw cycles between −80 °C and 28 °C to release intracellular virus. Subsequently, the mixture was centrifuged at 12,000 × g for 10 min at 4 °C. The supernatant was collected and used as the viral inoculum for the next passage. A portion of the supernatant was collected for RNA extraction and viral copy number analysis. Second generation (P2): Fresh cells of the same cell line were seeded into 35 mm culture dishes, allowed to adhere overnight, and then infected with the viral supernatant collected from P1. The remaining steps were identical to those used for P1. Samples were collected at 72 hpi and processed as described above to obtain the viral supernatant for the next passage. Third generation (P3): The same procedure was repeated. Fresh cells were infected with the viral supernatant obtained from P2, and samples were collected at 72 hpi.

### 2.5 Quantitative detection of viral copy number

Cell samples lysed with TRIzol were removed from −80 °C storage and thawed at room temperature. Chloroform (200 μL) was added to each tube. Samples were vigorously shaken for 15 s and incubated at room temperature for 5 min. These mixture was then centrifuged at 12,000 × g for 15 min at 4 °C. The upper aqueous phase was carefully aspirated into a new RNase-free centrifuge tube. An equal volume of isopropanol was then added, and samples were mixed by inversion and incubated at room temperature for 10 min. After centrifugation at 12,000 × g for 10 min at 4 °C, the supernatant was discarded. The RNA pellet was washed twice with 75% ethanol (prepared with DEPC water). Each wash was followed by centrifugation at 7,500 × g for 5 min at 4 °C. After removal of the ethanol, the pellet was air-dried at room temperature for 5–10 min and dissolved in 50 μL DEPC-treated water.

RNA was extracted from viral supernatant samples using the TIANGEN Viral RNA Extraction Kit (Tiangen Biotech, Beijing, China) according to the manufacturer’s instructions. Briefly, 140 μL of viral supernatant was mixed with 560 μL of lysis buffer and incubated at room temperature for 10 min. Subsequently, 560 μL of absolute ethanol was added. The mixture was transferred to an adsorption column, centrifuged at 12,000 × g for 1 min, and the flow-through was discarded. Protein-removal solution and wash solution was added sequentially according to the manufacturer’s protocol. RNA was then eluted with 50 μL RNase-free ddH₂O. The extracted RNA was either used immediately or stored at −80 °C.

The viral copy number was quantified using the CMNV real-time fluorescent quantitative RT-PCR (RT-qPCR) detection kit co-developed by Qingdao Lijian Biotechnology Co., Ltd. and the Yellow Sea Fisheries Research Institute, Chinese Academy of Fishery Sciences. This kit was designed using specific primers and TaqMan probes that target a conserved region of the CMNV capsid protein gene. A 20 μL reaction mixture was prepared according to the manufacturer’s instructions, and 5 μL of template was added. The thermal cycling program consisted of 50 °C for 5 min, pre-denaturation at 95 °C for 30 s, followed by 40 cycles of denaturation at 95 °C for 10 s and annealing/extension at 60 °C for 30 s. Each sample was analyzed in triplicate. Absolute viral copy number were calculated from a standard curve, and the mean value was used for the final viral copy number analysis.

### 2.6 Observation of cytopathic effect (CPE)

During viral infection and serial passage, cell morphology was observed daily under an inverted microscope (IMT-2, Olympus, Japan). The occurrence of CPE, including cell rounding, shrinkage, detachment, and lysis, was recorded. Images were captured at 72 hpi for each passage to compare cell morphology before and after infection. Three replicate wells were examined for each cell line, and all observations were recorded.

### 2.7 Viral replication efficiency assay

EPC, TiB, and S2 cells were seeded into 35 mm cell culture dishes (5 × 10^5^ cells/dish) and incubated overnight under their respective culture conditions to allow cell attachment. The medium was then replaced with maintenance medium containing 2% FBS. Prior to infection, cells from each cell line were counted to ensure consistent cell numbers per dish across all cell lines. The experimental group was infected with CMNV at an MOI of 0.001. After infection, cells were incubated at 28 °C (with or without CO_2_ depending on cell type) for 2 h to allow viral adsorption. The inoculum was then discarded, and the cells were washed three times with PBS to remove unbound virus particles. Fresh maintenance medium was added, and cells were further cultured. One set of samples was collected at 2 hpi (immediately after washing, serving as the initial input control), while the remaining samples were collected at 24, 48, and 72 hpi. Three replicate dishes were included for each time point. At collection, the supernatant and cells were thoroughly mixed and transferred to 15 mL centrifuge tubes. The samples were subjected to three freeze–thaw cycles between −80 °C and 28 °C to release intracellular virus (all samples were processed identically, so systematic bias does not affect intergroup comparisons), and subsequently centrifuged at 12,000 × g for 10 min at 4 °C. The supernatant was collected for viral RNA extraction. Viral copy numbers were quantified by RT-qPCR and expressed as fold increase relative to the initial input (2 hpi).

### 2.8 Cell viability assay by CCK-8

EPC, TiB, and S2 cells were seeded into 96-well plates (3 × 10^4^ cells/well) and incubated overnight under their respective culture conditions to allow cell attachment. The old medium was then aspirated and replaced with maintenance medium containing 2% FBS. The experimental group was infected with CMNV at an MOI of 0.001, the mock group received an equal volume of CMNV-negative shrimp extract, and the blank control group received an equal volume of PBS. A background blank group (medium only, without cells) was also included for background subtraction. Each group included four replicate wells. Cell viability was assessed at 24, 48, and 72 hpi. At each time point, 10 μL of CCK-8 solution was directly added to each well (to a final concentration of 10%, in a total volume of 100 μL) without removing the culture supernatant. The plates were then incubated at 28 °C in the dark for 2 h. Following incubation, the absorbance at OD_450_ was measured using a microplate reader.

### 2.9 Statistical analysis

All experimental data processing and graphing were performed using GraphPad Prism 8 software. Viral load data are expressed as mean ± standard deviation (SD). Statistical analyses were performed using one-way or two-way analysis of variance (ANOVA) or multiple Welch’s t-tests with appropriate post-hoc corrections, as specified in the corresponding figure legends. The statistical significance level was set at 0.05.

## 3 Results

### 3.1 CPE of CMNV infection in eight cell lines at different time points

**Figure 1:**
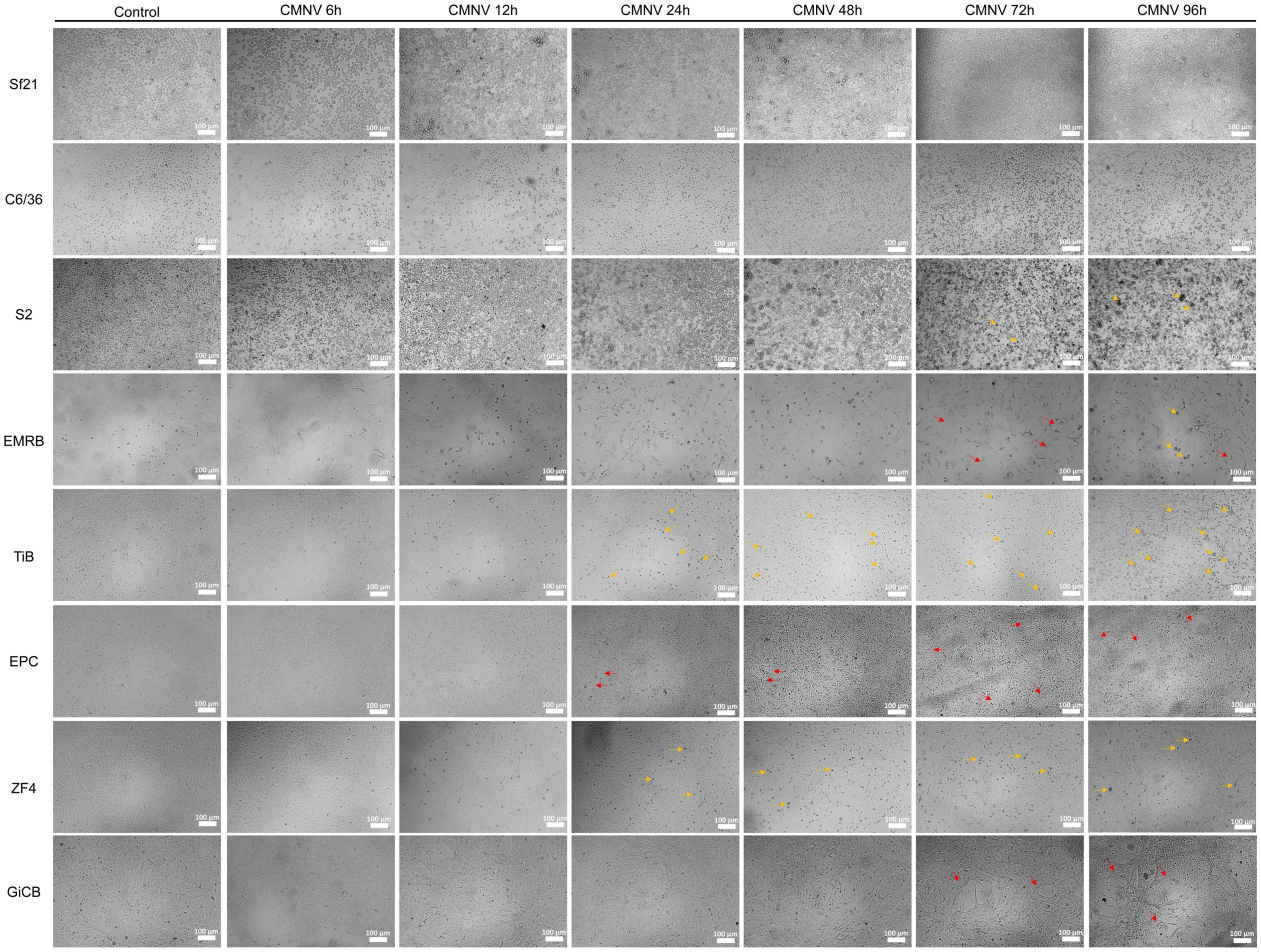
CPE of CMNV infection in eight cell lines at different time points. Cell lines (Sf21, C6/36, S2, EMRB, TiB, EPC, ZF4, and GiCB) were infected with CMNV and observed under an inverted microscope at 6-, 12-, 24-, 48-, 72-, and 96-hpi. Red arrows indicate plaques, and yellow arrows indicate dead cells.

Eight cell lines (Sf21, C6/36, S2, EMRB, TiB, EPC, ZF4, and GiCB) were infected with CMNV to identify susceptible cell lines. Cell morphology was photographed at different time points to monitor CPE. As illustrated in Figure 1, among the three insect cell lines (Sf21, C6/36, and S2), limited cell death was observed in S2 cells at 72 and 96 hpi, whereas no obvious CPE was observed in Sf21 and C6/36 cells within 96 hpi. In CMNV-infected EMRB cells, cell boundaries began to blur at 12 hpi. Limited cell death was observed, and a small number of plaques appeared at 72 and 96 hpi. TiB cells exhibited obvious cell death from 24 hpi. The number of dead cells gradually increased over time and reached a maximum at 96 hpi. However, no plaques were observed. In EPC cells, plaques appeared at 24 hpi and increased in number thereafter. In ZF4 cells, sporadic cell death began at 24 hpi and gradually increased over time. However, no obvious plaques were observed, and the number of dead cells was much lower than that of the TiB cells. In GiCB cells, plaques appeared at 72 and 96 hpi, but obvious cell death was not evident.

### 3.2 Replication of CMNV in different cell lines at different time points

**Figure 2:**
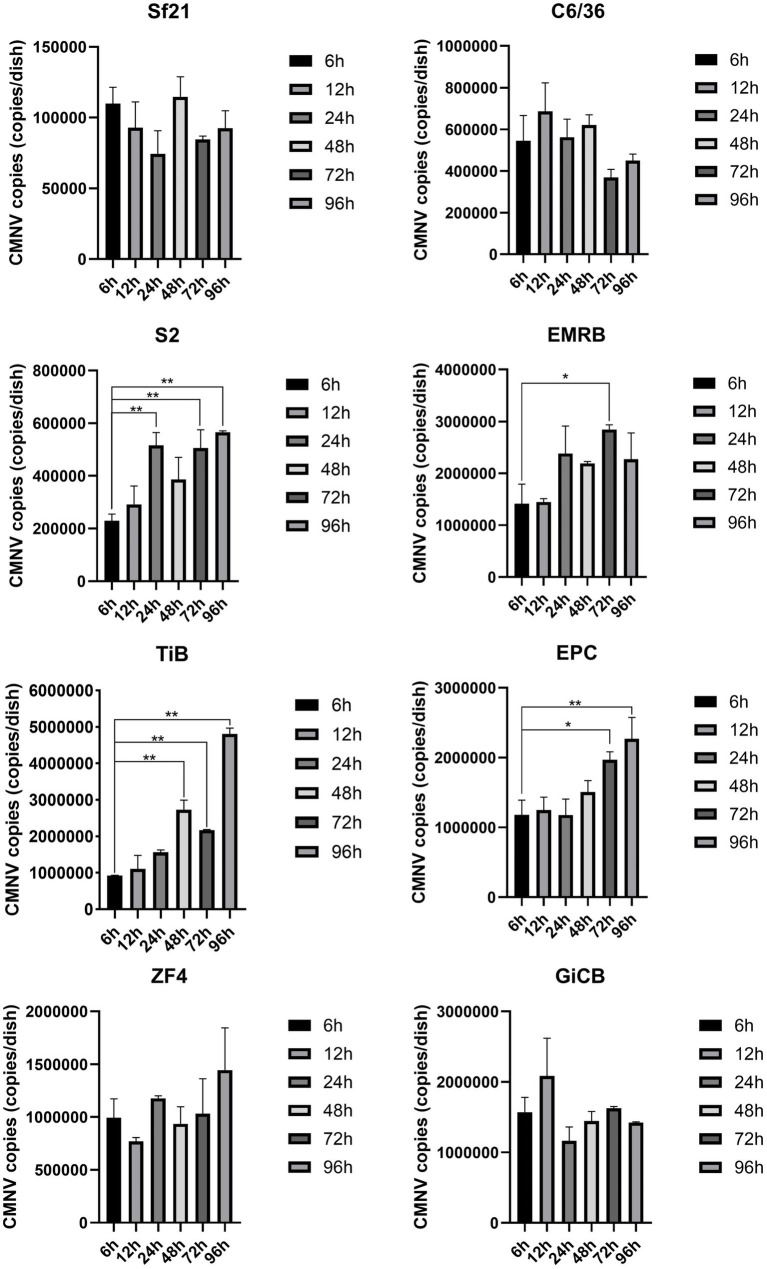
Replication kinetics of CMNV in eight cell lines. Eight cell lines (Sf21, C6/36, S2, EMRB, TiB, EPC, ZF4, and GiCB) were infected with CMNV. Viral RNA copy numbers in cell samples (cells only) were quantified by RT-qPCR at 6-, 12-, 24-, 48-, 72-, and 96-hpi. Data are presented as mean ± SD (*n* = 3). Statistical significance was determined by one-way ANOVA followed by Dunnett’s post-hoc test comparing each time point to 6 hpi. Only significant differences are indicated: *p < 0.05, **p < 0.01.

Cell samples were collected at different time points to determine the viral copy number (Figure 2). The results showed no significant differences in CMNV load among different time points in Sf21, C6/36, ZF4, and GiCB cells. In EMRB cells, viral load showed an upward trend over time, and a significant difference was observed between 6 and 72 hpi. However, no significant differences were detected between other sampling time points. In S2, TiB, and EPC cells, viral load increased significantly over time. Viral loads peaked at 96 hpi and were approximately 2.0- to 5.0- fold higher than those at 6 hpi. Among the eight cell lines, S2, TiB, and EPC cells exhibited substantial increases in CMNV load, followed by a modest increase in EMRB, whereas Sf21, C6/36, ZF4, and GiCB cells showed no significant changes throughout the observation period. The temporal patterns of viral replication generally corresponded with the severity of CPE observed in these cell lines.

### 3.3 Changes in CMNV-induced cytopathic effects during serial passage

**Figure 3:**
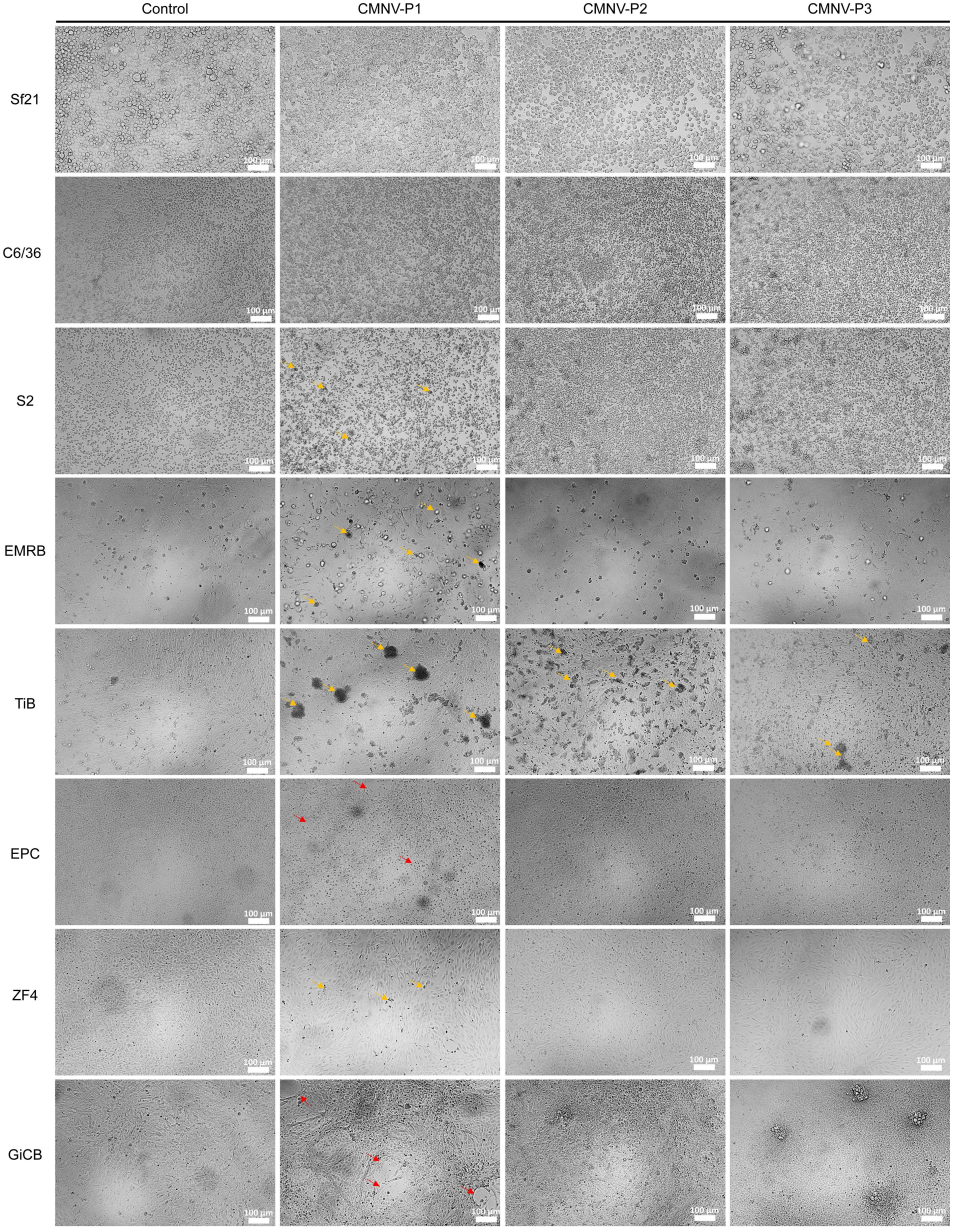
Cytopathic effects during serial blind passage of CMNV in eight cell lines. Three generations of blind passage were performed, and cell morphology was observed at 72-hpi for each passage. Red arrows indicate plaques, and yellow arrows indicate dead cells.

To assess CMNV replication during cell passage, a three-generation blind passage experiment was performed. CPE was continuously monitored in all eight cell lines (Figure 3). In the first passage (P1), limited cell death or plaque formation was observed in S2, TiB, EPC, ZF4, and GiCB cells. EMRB cells showed unclear cell boundaries and cell death, whereas no obvious morphological changes were observed in Sf21 and C6/36 cells. These results were consistent with those obtained during the initial infection experiment (Figure 1). In the second (P2) and third (P3) passages, only TiB cells showed cell death. No obvious morphological changes were observed in the other cell lines. Overall, the severity of CMNV-induced CPE decreased gradually with increasing passage number.

### 3.4 Analysis of CMNV proliferation during serial passage in different cell lines

**Figure 4:**
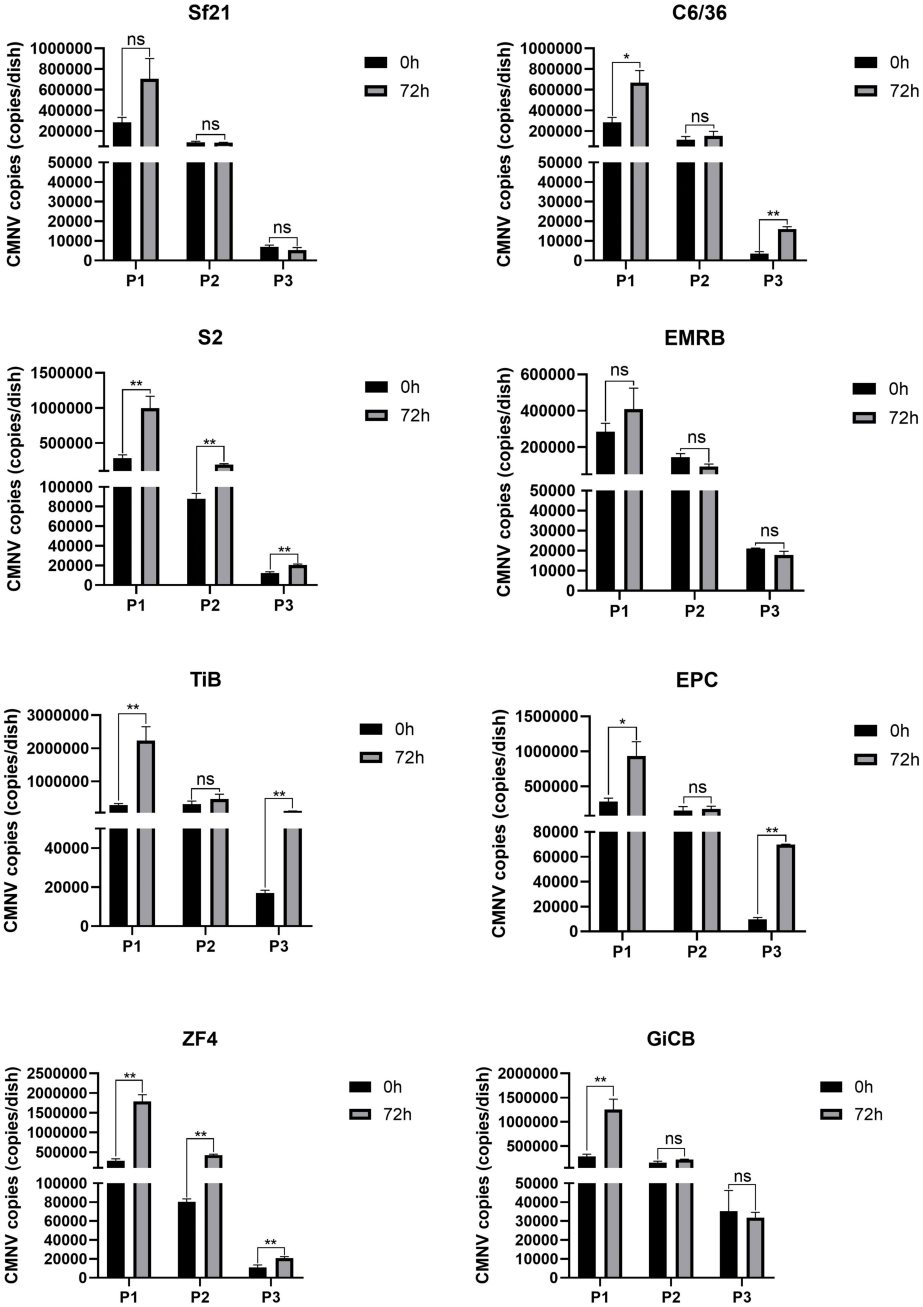
Replication of CMNV during serial blind passage in eight cell lines. Viral RNA copy numbers were quantified at 0 h and 72 h for each of the three passages (P1, P2, and P3). For each passage, 0 h samples were collected immediately after virus inoculation (representing the initial inoculum), while 72 h samples represent the total viral copies recovered from both cells and supernatant after 72 h of culture. Data are presented as mean ± SD (*n* = 3). Statistical significance comparing 72 h versus 0 h within each passage was determined by multiple *t*-tests (without assuming consistent SD) with Holm-Sidak correction for multiple comparisons. All *p*-values were adjusted for multiple comparisons. *p < 0.05, **p < 0.01; ns, not significant.

Changes in viral copy number during the three-generation blind passaging were quantified to evaluate the stability of CMNV replication in different cell lines (Figure 4). In P1, CMNV replicated to different degrees in all cell lines. Significant increases in viral RNA copies were observed in C6/36, S2, TiB, EPC, ZF4, and GiCB cells, with viral RNA copies approximately 1.5- to 8.0-fold higher than the initial inoculum. Specifically, in TiB cells, the viral RNA copies at 72 h was approximately 8-fold higher than that at 0 h (2.23 × 106 copies/dish versus 2.84 × 105 copies/dish). In P2, no significant change in CMNV RNA copy number was detected in Sf21, C6/36, EMRB, TiB, EPC, and GiCB cells. In contrast, CMNV continued to replicate and thus amplify in S2 and ZF4 cells. In ZF4 cells, the viral RNA copies at 72 hpi was approximately 5.0-fold higher than that at 0 h (4.25 × 10^6^ copies/dish versus 8.03 × 10^5^ copies/dish). In P3, CMNV RNA copies increased significantly in C6/36, S2, TiB, EPC, and ZF4 cells and were approximately 2.0- to 7.0-fold higher than the initial inoculum. In EPC cells, the viral RNA copies at 72 h was approximately 7-fold higher than that at 0 h (6.99 × 10^4^ copies/dish versus 9.75 × 103 copies/dish). No significant change in CMNV RNA copy number was detected in Sf21, EMRB, and GiCB cells. Overall, S2 and ZF4 cells showed significant increases in CMNV RNA copies across all three passages.

Based on the combined results of CPE observations and viral RNA replication across the eight cell lines, three cell lines-EPC, TiB, and S2-were selected for further characterization of viral replication efficiency and effects on cell viability, as they exhibited both obvious CPE and sustained viral RNA replication through at least two passages. Notably, ZF4 and C6/36 supported viral RNA replication across multiple passages without obvious CPE, and were therefore tentatively designated as atypically susceptible.

### 3.5 Viral replication efficiency and cell viability of CMNV in EPC, TiB, and S2 cell lines

**Figure 5:**
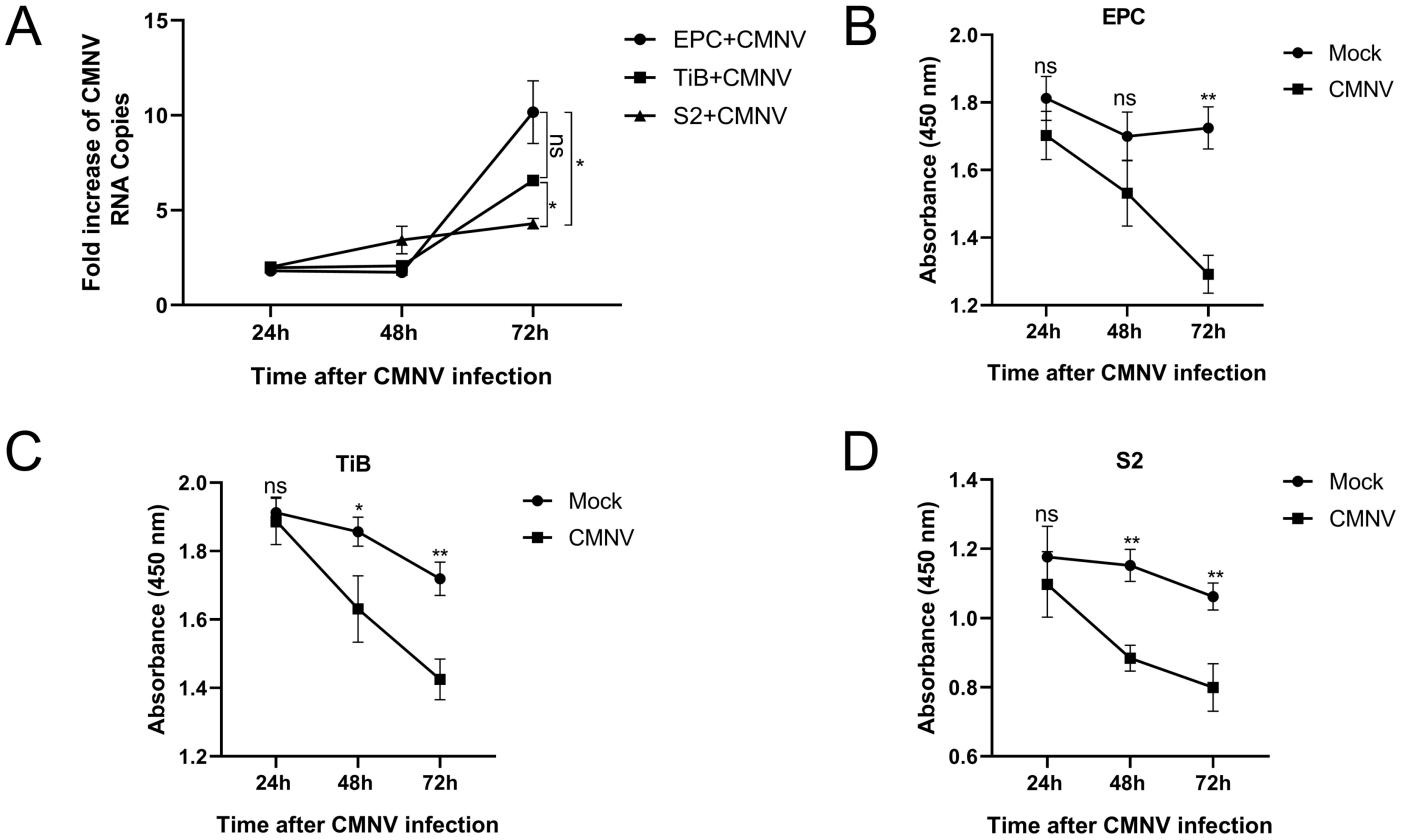
Replication kinetics and cell viability of CMNV in EPC, TiB, and S2 cell lines. **(A)** Viral RNA copy numbers, expressed as fold increase relative to the initial input at 2 hpi, were measured by RT-qPCR at 24-, 48-, and 72-hpi. Data are shown as mean ± SD (*n* = 3). Differences between group means were analyzed using two-way ANOVA with Tukey’s multiple comparisons test. *p < 0.05; ns, not significant. **(B–D)** Cell viability was measured by CCK-8 assay in mock- and CMNV-infected cells (MOI = 0.001) at the indicated time points. Data are shown as mean ± SD (*n* = 4). Statistical significance between mock- and CMNV-infected groups at each time point was determined by multiple *t*-tests (without assuming consistent SD) with Holm-Šídák correction for multiple comparisons.*p < 0.05, **p < 0.01; ns, not significant.

To evaluate the replication efficiency of CMNV in EPC, TiB, and S2 cells, the viral titer of the inoculum was first determined in each cell line using the TCID_50_ endpoint dilution assay, with cytopathic effects visualized by crystal violet staining (Supplementary Figure 1). Subsequently, all three cell lines were infected at a standardized multiplicity of infection (MOI = 0.001) to enable direct comparisons of viral replication kinetics and their impact on cell viability. As shown in Figure 5A, CMNV replication followed distinct kinetic profiles across the three cell lines. In EPC cells, viral RNA copy number peaked at 72 hpi, reaching a 10.2-fold increase over the initial input. TiB cells exhibited a similar peak time at 72 hpi, with a 6.6-fold increase, whereas S2 cells showed only a modest increase in copy number, with the fold change being significantly lower than that observed in the other two cell lines. The cytopathic effect of CMNV infection was further quantified by the CCK-8 cell viability assay (Figures 5B–D). In EPC cells, a significant reduction in cell viability was observed at 72 hpi relative to the mock-infected controls. In TiB cells, the reduction reached statistical significance at both 48 and 72 hpi. In S2 cells, a decreasing trend in cell viability was also noted at 48 and 72 hpi, although the magnitude of the effect was less pronounced. Overall, cell viability in all three cell lines declined progressively with the duration of CMNV infection, consistent with the establishment of productive infection and the associated cytopathic effects.

## 4 Discussion

In the present study, we employed RT-qPCR and CPE analysis in combination to evaluate CMNV infection across eight cell lines. RT-qPCR detects viral nucleic acids and thereby reflects viral genome replication within host cells, whereas CPE serves as a phenotypic indicator of viral replicative capacity and associated cytotoxicity. These two approaches provide complementary information at the molecular and cellular levels, respectively. However, each method has inherent limitations when used in isolation. RT-qPCR measures total viral RNA copies and cannot distinguish between infectious virions and non-infectious nucleic acids, such as defective particles, free RNA fragments, or residual input genomes (Mirmahdi et al., 2025). Similarly, the absence of CPE does not exclude active viral replication, as non-cytolytic or persistent infections may proceed without obvious cytopathic changes (Henle et al., 1958). Given these limitations, a single-round infection assay may not be sufficient to fully evaluate the replicative status of the virus in different cell lines. To address this, we performed a three-generation blind passage experiment. If the virus were merely residual or had undergone abortive infection, the nucleic acid signal would be progressively diluted out during serial passage. Conversely, if the viral copy number increased or was maintained across successive passages-even in the absence of obvious CPE-it would indicate that viral nucleic acid replication had occurred within the cells. Thus, the serial passage experiment served as a more rigorous dynamic approach to validate the authentic replicative capacity of the virus in the tested cell lines. Using this strategy, we identified three cell lines-EPC, TiB, and S2-that exhibited both obvious CPE and viral RNA replication in at least two passages. We further evaluated the replication efficiency of CMNV in these three cell lines at the RNA level under a standardized MOI.

Beyond detection methods, the nature of the viral inoculum itself warrants consideration. Another limitation of this study is that the viral inoculum consisted of crude CMNV extracts. We adopted this approach because high-titer CMNV-positive clinical samples are difficult to obtain. In addition, purification procedures such as sucrose-gradient or CsCl ultracentrifugation inevitably reduce viral recovery. Excessive virus loss could prevent subsequent cell infection experiments. Furthermore, the use of crude viral extracts is common during the initial establishment of cell culture systems for aquatic virology studies, particularly when no adapted cell line is available. Several studies have successfully used crude virus extracts to infect cultured cells and achieve viral propagation (Miao et al., 2000; Sudhakaran et al., 2007). Nevertheless, crude inoculum may contain host-derived cytotoxic factors. These components may have contributed to the CPE observed during the initial passages. To evaluate this possibility, we prepared tissue extracts from CMNV-negative healthy shrimp using the same procedure as for the virus inoculum, and used them as a mock control to assess the cytotoxicity of host-derived components in three susceptible cell lines (EPC, TiB, and S2). Our results demonstrated that CMNV remained the primary factor responsible for the CPE observed in the sensitive cell lines, while host-derived factors played only a minor role and affected semi-adherent cells such as S2 to a limited extent (Supplementary Figure 2).

Beyond these methodological considerations, our serial passage experiments also revealed several biologically intriguing phenomena. Notably, during serial passage, we observed that CMNV infection of ZF4 and C6/36 cells did not induce obvious CPE. However, in the three-generation blind passage, ZF4 cells exhibited a progressive increase in viral copy numbers from P1 to P3, with a particularly pronounced increase at P2 (approximately 5-fold). C6/36 cells showed detectable viral amplification at both P1 and P3. This phenomenon may be attributable to several factors. First, the cumulative exposure time of the virus to host cells during serial passage is substantially longer than that in the single-infection assay, providing the virus with additional replication cycles and opportunities for adaptive evolution. RNA viruses frequently acquire adaptive mutations during serial passage that enhance their replicative fitness in specific cellular environments, as documented for West Nile virus (Brinton, 1983) and Sindbis virus (Lee et al., 2002), all of which gained advantageous mutations during passaging. Second, as the virus establishes more efficient replication, progeny virus accumulating in the supernatant may infect the next generation of cells at a higher effective MOI, establishing a positive feedback loop of “replication–release–reinfection” that further amplifies replication levels in subsequent passages. These two mechanisms may operate synergistically-adaptive mutations provide the virus with the capacity for efficient replication within the host cell, while the increase in effective MOI supplies the driving force that sustains and amplifies this capacity over successive passages. To determine whether CMNV has acquired adaptive mutations during its propagation in ZF4 cells, we plan to perform whole-genome sequencing on the virus harvested from P3 ZF4 cultures. This analysis will help elucidate the molecular basis by which CMNV establishes sustained replication in cell lines that do not exhibit obvious CPE.

The absence of CPE in ZF4 and C6/36 cells also prompted us to examine more closely the implications of CPE loss during serial passage. In the three-generation blind-passage experiment, CMNV nucleic acid amplification remained detectable in both P2 and P3. However, obvious cell death or plaque formation was absent. This phenomenon presents considerable difficulty for determining viral TCID_50_ using CPE as the readout. Several factors may account for this observation. First, the inoculum used for P2 and P3 was directly derived from the supernatant of the preceding passage without any concentration step, resulting in a progressive decline in total viral load with each successive transfer. Consequently, the viral titer may have dropped below the threshold required to elicit visible CPE. This interpretation is consistent with the principle of endpoint dilution assays such as TCID_50_, in which CPE becomes undetectable once the virus concentration falls below a critical level (Lei et al., 2021). Second, the gradual attenuation of CPE during serial passage may also reflect a transition of the virus from a lytic to a persistent non-cytolytic infection mode. Such a shift is not uncommon among RNA viruses; for instance, alphaviruses have been shown to switch between lytic and non-lytic replication strategies, thereby sustaining long-term replication without destroying host cells (Villanueva Guzman et al., 2026). This transition is likely facilitated by the intrinsically low pathogenicity of CMNV at both the cellular and tissue levels. Field observations indicate that CMNV causes less severe disease than other shrimp viruses, such as IMNV, and does not typically induce rapid host mortality or acute cell necrosis (Yao et al., 2022). This intrinsically attenuated virulence may provide a biological basis for the virus to shift toward a non-cytolytic infection mode during serial passage. Given the pronounced cross-species transmissibility of CMNV, we propose that the establishment of persistent non-cytolytic infection may represent one of the key mechanisms facilitating its long-term coexistence with multiple host species in nature. Persistent infection is increasingly recognized as a metastable state in virus–host coevolution, representing a widespread strategy that confers survival benefits to both viruses and their hosts at the population level (Goic and Saleh, 2012).

## 5 Conclusion

We systematically evaluated the susceptibility of eight cell lines to CMNV and identified multiple insect and fish cell lines that support CMNV replication at the RNA level and serial passage. We also characterized the relationship between viral replication and cytopathic effects in these cell lines. The identified cell culture system will facilitate future studies of CMNV pathogenesis, viral gene function, screening of antiviral compounds, and diagnostic development. More importantly, they provide an experimental platform for investigations of CMNV host adaptation and cross-species transmission. These findings establish a foundation for future studies of the molecular mechanisms that underlie CMNV infection and its emerging zoonotic potential.

## Data Availability

The original contributions presented in the study are included in the article/Supplementary material, further inquiries can be directed to the corresponding author.

## Supplementary material

SUPPLEMENTARY FIGURE 1 TCID50 Determination of CMNV in EPC, TiB, and S2 cell lines.

SUPPLEMENTARY FIGURE 2 Mock-controlled infection assay confirms virus-specific CPE of CMNV in EPC, TiB and S2 cell lines. The control group was inoculated with 50 μL of PBS. The mock group received an equal volume of tissue crude extract from CMNV-negative healthy shrimp. The CMNV group received tissue crude extract from CMNV-positive shrimp. Cells were fixed and stained with crystal violet at 72 hours post-infection. Red arrows indicate plaques formed as a result of virus infection.
