# Supplementary material for: Insect and fish cell lines support replication and serial passage of covert mortality nodavirus
Source: Front Microbiol. 2026 Aug 5;17:1858574. doi: 10.3389/fmicb.2026.1858574 (PMC13485972; doi:10.3389/fmicb.2026.1858574)
Supplement: Supplementary file 1 [file Supplementary_file_1.docx]

Insect and fish cell lines support replication and serial passage of covert mortality nodavirus

**Zhaoxi Wang, Zhanqing Sun, Tingting Xu, Na Wang, Qingli Zhang**

# *Supplementary Material*

# 1 Supplementary methods description

## 1.1 TCID_50_ Determination

EPC, TiB, and S2 cells were seeded into 96-well plates (Corning, USA) and incubated overnight under their respective culture conditions to allow cell attachment. The old medium was then aspirated and replaced with maintenance medium containing 2% FBS. The CMNV stock was serially diluted 10-fold (from 10^-1^ to 10^-6^) with maintenance medium, yielding six dilution levels. A volume of 100 μL of each virus dilution was added to the cell monolayers, with four replicate wells per dilution (preliminary experiments confirmed that this dilution range adequately covered the endpoint dilution). A blank mock group (CMNV-negative shrimp extract) was also included. After infection, cells were incubated at 28℃ (with or without CO_2_ depending on cell type) for approximately 72 h. The supernatant was then discarded, and the cells were washed three times with PBS, fixed with 4% paraformaldehyde for 20 min, washed three times with PBS, stained with 0.1% (w/v) crystal violet for 10 min, washed three times with PBS, air-dried, and photographed. The number of wells exhibiting CPE at each dilution was recorded, and the TCID_50_ of CMNV in each cell line was calculated according to the Reed-Muench method.

## 1.2 Preparation of mock inoculum

For the mock control, healthy shrimp tissue that tested negative for CMNV by qPCR was selected, and CMNV-negative shrimp extract was conducted following the procedures described in the "Virus preparation" section of Materials and Methods.

## 1.3 Mock-controlled infection assay

EPC, TiB, and S2 cells were seeded into 24-well plates at a density of approximately 1×10^5^ cells/well and incubated overnight to allow cell attachment. Before infection, the old medium was aspirated and replaced with maintenance medium (infection medium) containing 2% FBS. Subsequently, 50 μL of virus extract was added to each well, with CMNV-negative shrimp extract serving as the mock group, CMNV-positive shrimp extract as the positive group, and an equal volume of PBS as the blank control group. Each group included four replicate wells. At 72 h post-infection, the supernatant was carefully aspirated, and the cells were washed three times with PBS. The cells were then fixed with 4% paraformaldehyde for 20 min, followed by three washes with PBS. The fixed cells were stained with 0.1% (w/v) crystal violet for 10 min, washed three times with PBS, air-dried, and photographed for documentation.

# 2 Supplementary results

## 2.4 TCID_50_ determination of CMNV in EPC, TiB, and S2 cell lines

Under the experimental conditions of this study, the viral titer of CMNV in EPC cells was 10^3.5^ TCID_50_/0.1 mL, and in TiB and S2 cells, it was 10^2.3^ TCID_50_/0.1 mL (Supplementary Figure 1).

## 2.5 Confirmation of CMNV-Specific CPE using mock-controlled infection assay

In EPC cells, the CMNV-infected group exhibited obvious plaque formation, whereas no significant differences were observed between the mock group and the control group, indicating that the CPE in EPC cells are virus-specific. In TiB cells, compared with the mock group, the CMNV-infected group showed notably lighter crystal violet staining and a small number of plaques, suggesting that CMNV infection caused damage to the cell monolayer; no obvious differences were observed between the mock group and the control group, further supporting the conclusion of CMNV-specific cytopathogenicity. Regarding S2 cells, it should be particularly noted that this cell line exhibits a semi-adherent growth phenotype under static culture conditions, and its inherent attachment ability is relatively weak. Consequently, the overall staining intensity in all groups was lower than that observed in EPC and TiB cells. In S2 cells, both the mock group and the CMNV-infected group showed fewer adherent cells compared with the control group, suggesting that host-derived extracts had some impact on cell growth. However, the number of residual adherent cells in the CMNV-infected group was even lower than that in the mock group, indicating that CMNV infection exerted an additional inhibitory effect on S2 cell growth beyond that of host-derived factors alone. In summary, although host-derived tissue extracts showed a detectable effect on the growth of S2 cells, the mock groups in both EPC and TiB cells exhibited no obvious CPE. These results demonstrate that CMNV is the primary factor responsible for the CPE observed in the sensitive cell lines, while host-derived factors play only a minor role and may influence semi-adherent cells such as S2 to a limited extent (Supplementary Figure 2).

# 3 Supplementary Figures and Tables

## 3.6 Supplementary Figures

## Supplementary Figure 1. TCID_50_ Determination of CMNV in EPC, TiB, and S2 cell lines.

**Supplementary Figure 2.** Mock-controlled infection assay confirms virus-specific CPE of CMNV in EPC, TiB and S2 cell lines.The control group was inoculated with 50 μL of PBS. The mock group received an equal volume of tissue crude extract from CMNV-negative healthy shrimp. The CMNV group received tissue crude extract from CMNV-positive shrimp. Cells were fixed and stained with crystal violet at 72 hours post-infection. Red arrows indicate plaques formed as a result of virus infection.
